# NORSE/FIRES: how can we advance our understanding of this devastating condition?

**DOI:** 10.3389/fneur.2024.1426051

**Published:** 2024-08-08

**Authors:** Dimitrios Champsas, Xushuo Zhang, Richard Rosch, Evangelia Ioannidou, Kimberly Gilmour, Gerald Cooray, Gavin Woodhall, Suresh Pujar, Marios Kaliakatsos, Sukhvir K. Wright

**Affiliations:** ^1^Department of Neurology, Great Ormond Street Hospital (GOSH), London, United Kingdom; ^2^Institute of Health and Neurodevelopment, School of Health and Life Sciences, Aston University, Birmingham, United Kingdom; ^3^Department of Clinical Neurophysiology, King’s College Hospital London NHS Foundation Trust, London, United Kingdom; ^4^Departments of Neurology and Pediatrics, Columbia University, New York, NY, United States; ^5^Department of Immunology, Great Ormond Street Hospital (GOSH), London, United Kingdom; ^6^Biomedical Research Centre (BRC), London, United Kingdom; ^7^Institute of Child Health, University College London, London, United Kingdom; ^8^Department of Neurophysiology, Great Ormond Street Hospital (GOSH), London, United Kingdom; ^9^Clinical Neuroscience, Karolinska Institutet, Stockholm, Sweden; ^10^Birmingham Women’s and Children’s Hospital NHS Trust, Birmingham, United Kingdom

**Keywords:** NORSE, FIRES, status epilepticus, immunomodulation, autoantibodies, animal models

## Abstract

**Introduction:**

New onset refractory status epilepticus (NORSE) is a rare and devastating condition characterised by the sudden onset of refractory status epilepticus (RSE) without an identifiable acute or active structural, toxic, or metabolic cause in an individual without a pre-existing diagnosis of epilepsy. Febrile infection-related epilepsy syndrome (FIRES) is considered a subcategory of NORSE and presents following a febrile illness prior to seizure onset. NORSE/FIRES is associated with high morbidity and mortality in children and adults.

**Methods and results:**

In this review we first briefly summarise the reported clinical, paraclinical, treatment and outcome data in the literature. We then report on existing knowledge of the underlying pathophysiology in relation to *in vitro* and *in vivo* pre-clinical seizure and epilepsy models of potential relevance to NORSE/FIRES.

**Discussion:**

We highlight how pre-clinical models can enhance our understanding of FIRES/NORSE and propose future directions for research.

## Introduction

The term NORSE (New Onset Refractory Status Epilepticus) (1) was first used in 2005 to describe 7 cases (females, mean age 33 yrs., range 20–52 yrs) of status epilepticus (SE) refractory to standard treatment protocols with a poor outcome (5 died, 2 were in vegetative state) in whom a previous history of epilepsy or underlying cause was not identified. Five year later, “FIRES” (Febrile Infection-Related Epilepsy Syndrome) emerged as a clinical entity describing a case-series of 22 previously typically developing children (3–15 yrs) with prolonged or recurrent seizures occurring ~5 days after fever onset (2, 3). In 2018, an expert group released a Consensus definition of New-Onset Refractory Status Epilepticus (NORSE) (4) as a clinical presentation, not a specific diagnosis, in a patient without active epilepsy or other pre-existing relevant neurological disorder, with new onset of refractory status epilepticus (RSE) without a clear acute or active structural, toxic, or metabolic cause but including patients with viral or autoimmune causes. FIRES was designated a subtype of NORSE where, following a minor febrile illness, seizures increase rapidly (100 s/day) and worsen to SRSE (4). Previously the term FIRES was used exclusively in children, however, it is now accepted that children can have NORSE and adults can have FIRES.

Despite progress in the clarification of NORSE/FIRES as a clinical syndrome, understanding of the underlying pathogenic mechanisms is limited. A cryptogenic cause accounts for up to 50% (5) of cases with these cases reported to have worst outcomes (6).

Treatment of FIRES/NORSE is challenging, and traditional antiseizure medicines (ASMs) used in status epilepticus often prove ineffective against the unrelenting ictal activity (7). All the available evidence for NORSE/FIRES treatment is from case-series, there have been no systematic clinical trials to date. As positive results from therapies are more likely to be published, the bias in treatment responsiveness needs acknowledgement. A Delphi expert consensus on a suggested treatment algorithm was recently published (2) stating initial treatment of SE and RSE with ASMs and anesthetic drugs as per published and local guidelines, and management of possible infections alongside diagnostic work-up and treatment for etiology if identified. If there is incomplete response then initiation of first-line immunological treatment (steroids, IVIG, PLEX) within the first 72 h of seizures is recommended. If ongoing unresponsiveness to treatment, children (and adults if possible) can be started on the ketogenic diet, followed by second-line immunological treatment [including IL-1Ra and IL-6 antagonists (if cryptogenic), and Rituximab (if neuronal autoantibody identified or autoimmune encephalitis suspected)]. Other treatments published in case reports and small case series include intrathecal dexamethasone (8, 9), cannabidiol (10, 11), and Janus kinase (JAK) inhibitors (12). Further studies are needed to determine the effectiveness of these treatments.

There is increasing interest in non-pharmacological treatment of SE in NORSE/FIRES, including Vagal Nerve Stimulation (VNS), deep brain stimulation (DBS) and electroconvulsive therapy (ECT). In a recent systematic review (13, 14), 20 patients with NORSE/FIRES treated with neuromodulation were identified. VNS was used in seven patients with cessation of SE in five, although two died. DBS was implanted in four patients (in one VNS first and then DBS), all achieving a good outcome for SE. ECT was performed in 10 patients, of which three were under 4-years-old with a diagnosis of FIRES (13). In 9 out 10 patients SE resolved after 4–8 sessions. Of the 17 patients that survived, 11 had continuing epilepsy, 12 had cognitive and/or motor dysfunction and one patient remained in a vegetative state. In rare cases, dependent on etiology, epilepsy surgery including focal resection may be indicated (15, 16).

Outcomes remain frustratingly poor in children and adults with NORSE/FIRES. Mortality is approximately 12% in children and higher in adults (up to 30%) (5, 6, 17). In a recent prospective study, only 25% of children with FIRES recovered baseline functions compared to 89% of patients with refractory status epilepticus (18). Another long-term follow-up study of 48 adult patients found only 4% of patients made a full neurological recovery (19). In terms of seizure outcomes a recent systematic review (including 280 adult and 587 pediatric cases) found refractory epilepsy persisted in 40% of adults and nearly 60% of children (20). Long-term cognitive data was available in approximately 70% of pediatric cases and up to one third were described as moderately or severely disabled. Overall, the majority of patients did not return to a pre-morbid level of functioning (20).

There is an urgent need to develop pre-clinical models to understand the pathogenesis, and test effectiveness of specific therapies to improve outcomes in this catastrophic and life-changing condition.

**Figure 1:**
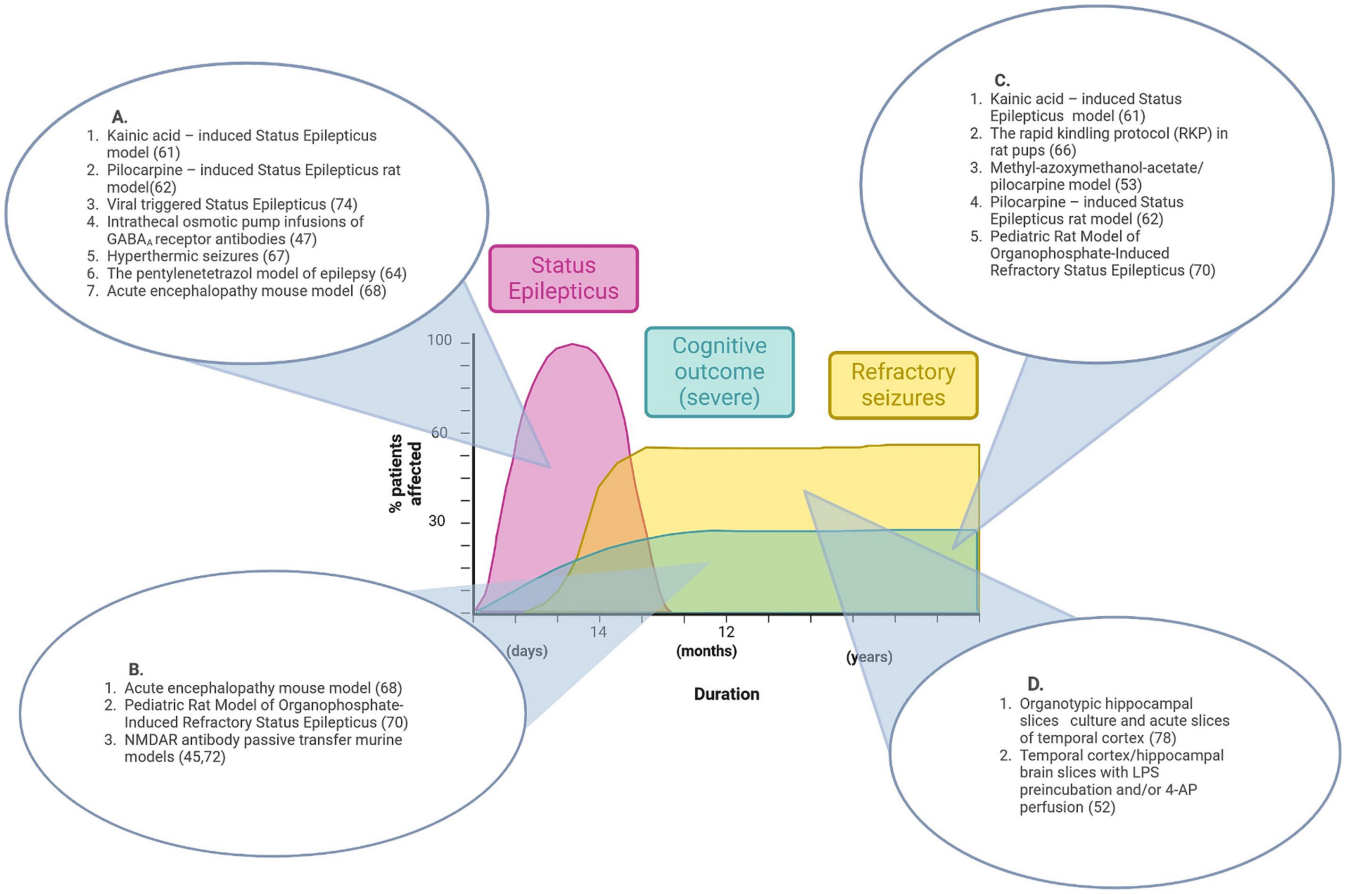
Graphical representation of features of pre-existing epilepsy animal models that align with disease stages of NORSE/FIRES. No single model recapitulates the full disease course. **(A)** Animal models that reproduce the acute phase of Status Epilepticus. **(B)** Animal models that show features of acute seizures and cognitive impairment. **(C)** Animal models that show features of both refractory seizures and cognitive impairment. **(D)** Pre-clinical models that demonstrate refractory seizures *in vitro*. Figure created in Biorender.com.

In this review, we examine the potential relevance of existing pre-clinical seizure/epilepsy models and SE study approaches to NORSE/FIRES pathogenesis (Figure 1; Table 1) and propose future research directions.

**Table 1:** This table summarizes *in vivo* and *in vitro* seizures models, with specification of species, features, and relevance with NORSE and FIRES for each individual model.

*In vivo* model	Epilepsy/seizure induction method	Species	Features of the model	Relevance with NORSE/FIRES
Kainic acid – induced status epilepticus (SE) model (21)	Intracranial (intra-amygdala/intrahippocampal) microinjection	8-week-old C57BL/6 mice	Intra-amygdala more severe SE and higher mortality (44%) than intrahippocampal model. Absent or short latent period from SE to spontaneous recurrence seizures	Prolonged (12–18 h) SE leading quickly to spontaneous recurrence seizures (SRS)
Pilocarpine – induced SE rat model (22)	A single acute dose of pilocarpine (380 mg/kg, i.p.) injected to induce SE	Adult male Wistar rats	One of the most prevalent models of human temporal lobe epilepsy, which exhibits physiological, behavioral, electroencephalographic and seizure patterns resembling those of temporal lobe epilepsy	Pilocarpine model has upregulation of CCR2 and C-C motif chemokine ligand 2 (CCL2) within glial and neuronal cells in the hippocampus, while NORSE has elevated levels of IL-6, TNF-α, IL-2, and IL-4 in the CSF, and elevated levels of IL-6 and TNF-α in the periphery (23). The innate immune system neuroinflammation is similar between NORSE/FIRES and pilocarpine model.
The pentylenetetrazol model of epilepsy (24)	Pentylenetetrazol intraperitoneally injected	Adult male Wistar rats	Pentylenetetrazol can be used to develop both acute and chronic models of epilepsy.	Relevant to resistant epilepsy (25)
The rapid kindling protocol (RKP) in rat pups (26)	Electrical stimuli to precipitate seizures with Saline or LPS (50 μg/kg i.p.)	Wistar rats pups (postnatal D14)	LPS increased the number of severe seizures and the mean duration of the seizures compared to the saline control group	LPS increased hippocampal baseline excitability, this can be reversed by IL-1 receptor antagonist, similar to drug effects in some patients with NORSE/FIRES. Neuroinflammation worsens outcome in this model of SE.
Hyperthermic seizures	Rats: 200 μg/kg LPS, 2.5 h after being placed in a 30°C incubator, ≥ 41.5°C induced seizures and ≥ 39.0°C was maintained for 30 min; mice: 100 μg/kg LPS, 2 h before hyperthermic seizures induction (27)	P14 Long Evans rats; P14 C57Bl/6 mice	LPS increases the rodents’ susceptibility to hyperthermic seizures; Priming LPS before seizures induction increases cytokine production and microglia activation; LPS also decreases the temperature threshold in seizures induction	Prolonged febrile seizures, cytokine production as seen in NORSE/FIRES.
Acute encephalopathy mouse model (28)	Low-dose LPS (50 or 100 mg/kg) intraperitoneally injected 2 h before hyperthermia treatment (41.5°C, 30 min).	P8 ICR mice of both genders	Exacerbation of BBB disruption; Microglia activation and small ischemic lesions in the cerebral cortexes were observed in some (29)	Displays symptoms of cytokine storm-induced acute encephalopathy.
Pediatric Rat Model of Organophosphate-Induced Refractory Status Epilepticus (30)	DFP (1.4x LD50 mg/kg, s.c.) administered to induce persistent SE. Animals were pretreated with pyridostigmine bromide (0.026 mg/kg, i.m.) 30 min before DFP injection.	Male Sprague–Dawley rats at postnatal day 21 (P21) (25–40 g).	Persistent refractory-like SE in P21 rats; progressive decline in learning and memory functions; progressive increase in anxiety-like symptoms and disruption in mood-related disturbances; progression of epileptogenesis with recurring spontaneous seizure episodes; widespread neurodegeneration of principal cells and inhibitory interneurons; persistent neuroinflammation with an extensive reactive microglia response; and aberrant mossy fiber sprouting in the hippocampus.	P21 rats are at a stage comparable to 2-to 4-year-old children. Features of behavioral change, neuropathology and inflammation consistent with FIRES/NORSE.
NMDAR antibody passive transfer rat model (31)	10 mg of NMDAR human monoclonal antibody, or 8 μL of NMDAR antibody or IgG (derived from patient’s plasma) via intracerebroventricular injection	Postnatal day 21 (P21) Wistar rats, male, with weights between 50–58 g	NMDAR antibodies cause spontaneous epileptiform activity including the sustained and repetitive hyperexcitable behavior (myoclonic twitches, jerks and jumps)	Identified etiology of NMDAR-Ab encephalitis in FIRES/NORSE patients (32)
IgG from anti-NMDAR encephalitis osmotic pump infusion (33)	Osmotic pump insertion to infuse polyclonal antibodies against the N-terminal domain of human GluN1protein (pooled from anti-NMDAR encephalitis and seizure patient)	male C57BL/6 mice that were 8–10 weeks old	CSF from patients with anti-NMDAR encephalitis induces seizures in mice, with 2% associated with only minimal behavior change	Identified etiology of NMDAR-Ab encephalitis in FIRES/NORSE patients (34)
Intrathecal osmotic pump infusions of GABA_A_ receptor monoclonal antibodies (mAbs) (35)	Cerebroventricular infusion with GABA_A_ receptor mAbs, in mice and rats	Male C57BL/6 mice, age:13–14 week; male Wistar rats at postnatal age of 21 days (weighing 50–58 g)	Spontaneous epileptiform activities including detectable ictal events. One died of status epilepticus with higher dose of infused antibody.	Refractive status epilepticus and GABA_A_R-Abs found in cases of NORSE/FIRES.
Viral triggered SE	Theiler’s murine encephalomyelitis virus injected intracerebrally (temporal region right hemisphere) to recapitulate epilepsy secondary to CNS viral infection	Male/female C57/BL6 mice from age 5-6 weeks	Acute and chronic seizures. Susceptible to handling-induced acute seizures.	Most observable seizures occur during the acute infection period of most relevance to initial phase of NORSE/FIRES, only a small proportion develop chronic spontaneous seizures (36).
Methyl-azoxymethanol-acetate/pilocarpine model (37)	Pregnant rats received 2 doses of methyl-azoxymethanol-acetate 12 h apart, at embryonic day 15. The young adult (2–3 months old litters) received pilocarpine treatment (270 mg/kg i.p.).	Pregnant Sprague–Dawley rats	Severe seizures with mortality in some. Severe CNS neuroinflammation with encephalitis-like features, brain malformation.	This model can be considered a model of autoimmune-associated epilepsy, and shows activation of adaptive immunity as seen in NORSE/FIRES (37).
*In vitro* model	Epilepsy/seizure induction method	Species	Features of the model	Relevance with NORSE/FIRES
Temporal cortex/hippocampal brain slices with LPS preincubation and/or 4-AP perfusion (38)	Brain slices preincubated for 30 min with the addition of 10 μg/mL lipopolysaccharide (LPS) before perfusion for 40 min with aCSF containing zero (0) Mg^2+^ and 100 μM 4-aminopyridine (4-AP)	28 to 30-day old male C57BL6/N mice	LPS exacerbates epileptiform activity in slices, reproducing the cytokine storm that precedes seizure precipitation in FIRES, with 4-AP inducing the refractive epileptiform activities	This model mimics the unresponsiveness of epileptiform events to antiseizure medications, as observed in NORSE/FIRES patients
Organotypic hippocampal slice cultures (39)	Isolated hippocampi culture from 350 μm slices.	P8 Sprague–Dawley rat pups	Progression in epileptiform activity, starting with interictal spike discharges around 14–17 days and transitioning to mostly ictal-like electrographic activity by day 25–30. Ictal events lasted greater than 3 min in duration in 80% of slices	Applicable to epileptogenesis in NORSE/FIRES (40)
4-aminopyridine (4-AP) model (41)	Rodent brain slices of 400 μm. 4-AP (100 μM) used for inducing seizure like events	Adult, 7–11-week-old, Wistar Han rats (200–350 g)	The model is generated via 4-AP, a non-selective potassium channel blocker and a potent proconvulsant compound in the limbic system	*In vitro* resistant seizures
Organotypic hippocampal slices culture and acute slices of temporal cortex (42)	0 Mg^2+^ exposure for 3 h at 28–30°C to hippocampal and temporal cortex cultures slices	GAD2-cre-tdTomato mice (C57BL/6 background, JAX lab) or Wistar rats; 7-day-old	With extended periods of 0 Mg^2+^, distinct ictal events are replaced by recurrent epileptiform discharges.	*In vitro* status epilepticus which strongly resemble clinical EEG recordings of convulsive status epilepticus.
Human brain tissue resected from epilepsy surgery patients (43)	None required	6 patients (F:M 9:7), median age 10.5 years (range 3–18 years)	Spontaneous *in vitro* epileptiform activity	Spontaneous epileptiform activity in human tissue from drug-resistant pediatric epilepsy patients; treatment response to KD (44)
Hippocampal slices (44, 45)	Superfusion of the high-K+ (8.5 mM) external solution	Wistar rats (postnatal days 9–19, weights from18 g – 49 g)	Epileptiform discharges in the stratum pyramidale of hippocampal slices	Tonic-firing pattern of spike events allowed reliable quantification of antiepileptic drugs to study focal seizures (46).
Intact cortical hippocampal formation (47)	Low-Mg^2+^ aCSF	Wistar rats at postnatal days 7 and 8	Spontaneous seizures were synchronized in hippocampal and cortical regions	Recurrent ictal-like events of infantile epilepsy that are resistant to commonly used antiepileptic drugs (47)

BBB, blood brain barrier; KD, ketogenic diet; LPS, lipopolysaccharide; SE, status epilepticus.

### Animal models of SE

In patients, when seizures in SE fails to respond to intravenous antiseizure medications (ASMs) it is termed refractory SE; super-refractory SE (SRSE), as seen in NORSE/FIRES, is defined when SE continues after 24 h or more of anesthetic treatment (48). Frequent EEG analysis is of paramount importance for treatment decisions and prognostication.

When considering animal models of SE, the onset of SE, severity and length varies according to animal strain, sex, age, species, and method of SE induction (49, 50) (Table 1). Quantification of SE severity is primarily based on behavioral assessments using established severity scales [e.g., the Racine scale (51)] not always reflecting underlying ictal EEG changes which are less frequently recorded – in one model it was found that 50% of behavioral seizures did not correspond with underlying epileptiform EEG activity (52–54).

EEG findings in patients with NORSE/FIRES often reveal a pattern of continuous and diffuse slowing, along with focal, multifocal or generalized epileptiform discharges (4, 17). Continuous EEG findings have also been reported (55) and identified common pathognomonic elements. In 6/7 patients a specific seizure pattern of focal faster waveforms, characterized as “sparks,” followed by a gradual appearance of well-formed rhythmic spike/spike and wave complexes was seen. “Ictal shifting” (shifting seizures with contralateral spread) was also described in 4 out of 7 patients (55). Given the significance of EEG in defining and mapping the evolution of SE and NORSE/FIRES, future animal models will need to provide this quantifiable data to ensure validity.

Recent work on EEG abnormalities in immune-mediated seizures has shown that computational models can explain observed EEG abnormalities and help identify putative pathophysiological mechanisms (56, 57). Dynamic Causal Modelling (DCM) of EEG data is an example of this computational approach that allows researchers to model the connectivity and interactions between brain regions, linking the physiology of integrated cortical circuits and their underlying synaptic constraints. DCM uses state-of-the-art Bayesian model inversion techniques to create *in silico* models that can be used to interrogate synaptic parameters that underlie specific dynamic brain states (58). In recent studies (56, 59), patient EEG recordings, *in vivo* animal experimental data, and *in silico* computational models have been combined to provide novel insights into the pathophysiology of immune-mediated epileptogenicity. In the context of NORSE/FIRES, such computational EEG approaches combining relevant pre-clinical model and patient data could be instrumental in identifying microscopic changes in the cortex that initiate seizure resistance and persistence phenotypical of NORSE/FIRES.

In NORSE/FIRES patients the most common neuropathology findings include neuronal loss, microglial activation and reactive gliosis (60, 61); 90% of children show brain abnormalities in long-term follow-up MRI scans, most commonly brain atrophy (74%, generalized in 58%) (62, 63). Given that many chemically or electrically-induced SE animal models go on to develop similar neuropathological changes following SE induction alongside chronic drug-resistant epilepsy and cognitive deficits, studying these long-term effects of NORSE/FIRES may be the most promising and relevant use of these models (64–66).

### Animal models of immune-mediated epilepsy and seizures

Serum and CSF studies for neuronal autoantibodies are the most commonly performed diagnostic examination after infection work-up in NORSE/FIRES cases (120/907; 61%) (32). Antibodies identified include anti-MOG (myelin oligodendrocyte protein), anti-NMDAR (N-methyl-D-aspartate receptor) antibodies, paraneoplastic and non-paraneoplastic (17, 32, 67–69). In adults with symptomatic NORSE autoimmune encephalitis is the most commonly identified cause (32). Previous studies in autoimmune encephalitis models have successfully used CSF and other human-derived samples to recapitulate core features of the disease including behavioral change, cognitive deficits and seizures (31, 70, 71). More recently, rodent models of immune-mediated seizures in the context of autoimmune encephalitis have also been developed proving the direct epileptogenicity of antigen-specific antibodies (e.g., to the NMDAR, GABA_A_R (gamma-aminobutyric acid receptor) and LGI1 (leucine-rich glioma inactivated 1) protein) (31, 35, 70). Unlike NORSE/FIRES, status epilepticus was not frequently seen and seizures resolve after completion of intracerebroventricular antibody infusion. This passive transfer approach may be challenging for establishing causality in NORSE/FIRES with no universal specific biomarker (72). However, if seizures were to occur on intracerebral application of CSF from NORSE/FIRES patients this may support the hypothesis of epileptogenic molecules being present in the human-derived samples, particularly as in a recent study pro-inflammatory cytokines/chemokines [e.g., IL-6 (interleukin-6), TNF-α (tumor necrosis factor-alpha), IL-8 (interleukin-8), CCL2 (C-C Motif Chemokine Ligand 2), MIP-1α (macrophage inflammatory protein-1 alpha), and IL-12p70 (interleukin 12p70)] in serum and CSF were found to be significantly increased in patients with SE compared with control patients without SE. In a subset analysis comparing cryptogenic NORSE patients (*n* = 51) and those with a known etiology for refractory SE (*n* = 47), only serum innate immunity pro-inflammatory cytokines/chemokines (CXCL8, CCL2 and MIP-1α) were significantly higher. Elevated innate immunity serum and CSF cytokine/chemokine levels in patients with NORSE were associated with worse outcomes at discharge and at several months after cessation of SE (73). It is important to note that in patients with increased CSF innate immunity cytokines, serial measurements frequently showed normalization of the values within days, and it is unclear if this is associated with immunomodulatory treatment. Further studies are needed with collection of acute and chronic CSF/serum samples and accurate documentation of immunotherapy and seizure frequency to establish cause and effect with respect to these immune mediators in NORSE/FIRES (67).

In animal models the activation and role of the innate immune system in the pathogenesis of status epilepticus has been shown in pre-clinical models and recently comprehensively reviewed by Vezzani et al. (74). In brief, there is strong evidence that neuronal cells release pro-inflammatory molecules after SE onset that initiate neuroinflammatory changes further influencing neuronal excitability and excitotoxicity (75). The fact that these changes are seen in normal rodent brains after SE induction is relevant to NORSE/FIRES and encouraging that anti-inflammatory treatments alter the progression to chronic epilepsy. A recently described *in vitro* model of FIRES using rodent brain slices and lipopolysaccharide (LPS) to induce inflammation demonstrated increased mRNA levels of relevant cytokines and inhibition of ictal activity with two specific immunomodulatory drugs, however the relatively acute time course of SE *in vitro* before treatment (10 min) and pre-incubation with these drugs may limit the translatability of the results to the clinical scenario (38).

Neuropathological findings in NORSE/FIRES also include perivascular T-cell infiltration, i.e., further involvement of the adaptive immune system beyond neuronal autoantibodies (60). A recent study highlighted the marginal involvement of adaptive immunity in the classical model of pilocarpine-induced epilepsy in normal rats when compared to their MethylAzoxyMethanol (MAM)/pilocarpine (MP) model that display malformed brains, status epilepticus and subsequent spontaneous recurrent seizures (37). This model of CNS autoimmune-associated epilepsy has potential to provide crucial insights into the complexities of the acute and chronic neuroimmunological response in NORSE/FIRES and the role of disease-modifying treatments.

Further work is needed to translate all these pre-clinical findings to NORSE/FIRES patients, specifically: the causal role of immune system mediators, identifying the optimal time in SE for immunodulatory intervention and if this intervention should be targeted to the innate and/or adaptive system.

### Genetic epilepsy models

In a recent systematic retrospective analysis of FIRES patients, genetic testing performed in 23 of 25 patients was non-diagnostic. Interestingly, when a broader cohort of 959 individuals with RSE was analyzed, the highest proportion of individuals with genetic RSE had onset within the first 3 months of life, whereas individuals with FIRES typically have onset in later childhood, with an age of onset ranging from 7.6 months to 18.7 years (69). Similar negative findings for a genetic etiology have been reported in previous studies (76, 77).

This lack of a single causative gene in NORSE/FIRES may hinder the development of a genetic epilepsy model but genetic therapy designed to suppress seizure activity, for example by modulating a specific channel (78), may be considered as a therapeutic approach that could be tested in pre-clinical models (79).

### Human brain tissue and epilepsy models

Human brain tissue is an invaluable resource that is being increasingly used in neuroscience research of direct relevance to NORSE/FIRES. For example, donated human tissue samples from pediatric epilepsy patients undergoing surgery have been used to demonstrate the therapeutic effectiveness of neurosteroids and decanoic acid, a major medium-chain fatty acid provided in the medium-chain triglyceride ketogenic diet, in *in vitro* electrophysiology studies (31, 43). Recent recommendations to integrate human tissue-based *in vitro* models into pre-clinical studies to develop anti-seizure therapies will benefit NORSE/FIRES research (44).

Using CSF and brain tissue samples, further insights into underlying immunopathology can be gained from single cell genomics as shown in pediatric epilepsy patients. Kumar et al., found in a study using samples from 6 pediatric epilepsy patients a pro-inflammatory microenvironment in the resected tissue, with extensive activation of microglia and infiltration of other pro-inflammatory immune cells (80). Of particular interest was clear demonstration of a direct interaction between T cells and microglia inside epileptic brain tissue similar to multiple sclerosis. One must caveat these findings with the knowledge that not all the transcriptional-level information will be translated to the protein level. Further validation is required before direct translation to management but nevertheless this advanced genomic analysis should be used in NORSE/FIRES to potentially identify relevant subpopulations of aberrant inflammatory and/or neuronal cells to investigate for pathogenicity.

To maximize the opportunities these rare samples can provide for research and increased understanding of NORSE/FIRES disease pathogenesis it is imperative that standardized procedures for specimen collection and biobanking are followed (67).

Figure 1 summarizes the contribution that existing pre-clinical epilepsy and seizure models could make to the understanding of NORSE/FIRES pathophysiology while also highlighting the research gap for a model that recapitulates the full clinical phenotype.

## Discussion and call to action

NORSE/FIRES is a devastating clinical syndrome characterized by acute intractable seizures evolving to treatment-resistant super-refractory status epilepticus, and mostly inevitable adverse cognitive impact in the long-term survivors. There is increasing worldwide collaboration in terms of disease definition, treatment guidelines and establishment of biorepositories underpinned by the unwavering support of dedicated affected family member advocates, parents, guardians, and carers. The role of an aberrant immune system response appears to be gaining evidence in terms of underlying pathophysiology and possible treatments. However, most of the published studies, case-reports and case series are comprised of small patient numbers and often retrospective in nature making it difficult to estimate treatment efficacy in the wider population. In this review we have highlighted how we can use existing pre-clinical models and human-derived samples to further NORSE/FIRES understanding as well as identifying promising future research areas. We call for experts from neurology, neurophysiology, computational biology, genetics, neuroscience, and immunology to collaborate and drive these promising areas of research forward to improve outcomes for affected patients.

## Author contributions

DC: Conceptualization, Writing – original draft, Writing – review & editing. XZ: Writing – original draft, Writing – review & editing. RR: Writing – original draft, Writing – review & editing. EI: Writing – original draft, Writing – review & editing. KG: Writing – original draft, Writing – review & editing. GC: Writing – original draft, Writing – review & editing. GW: Writing – original draft, Writing – review & editing. SP: Writing – original draft, Writing – review & editing. MK: Conceptualization, Writing – original draft, Writing – review & editing. SW: Conceptualization, Writing – original draft, Writing – review & editing.
